# Large-Scale MoS_2_ Pixel Array for Imaging Sensor

**DOI:** 10.3390/nano12234118

**Published:** 2022-11-22

**Authors:** Kang Liu, Xinyu Wang, Hesheng Su, Xinyu Chen, Die Wang, Jing Guo, Lei Shao, Wenzhong Bao, Honglei Chen

**Affiliations:** 1 State Key Laboratory of ASIC and System, School of Microelectronics, Zhangjiang Fudan International Innovation Center, Fudan University, Shanghai 200433, China; 2 School of Electronic Information, Soochow University, Suzhou 215006, China; 3 Shanghai Institute of Technical Physics, Chinese Academy of Sciences, Shanghai 200083, China

**Keywords:** molybdenum disulfide (MoS_2_), two-dimensional (2D) semiconductors, photo sensor

## Abstract

Two-dimensional molybdenum disulfide (MoS_2_) has been extensively investigated in the field of optoelectronic devices. However, most reported MoS_2_ phototransistors are fabricated using the mechanical exfoliation method to obtain micro-scale MoS_2_ flakes, which is laboratory- feasible but not practical for the future industrial fabrication of large-scale pixel arrays. Recently, wafer-scale MoS_2_ growth has been rapidly developed, but few results of uniform large-scale photoelectric devices were reported. Here, we designed a 12 × 12 pixels pixel array image sensor fabricated on a 2 cm × 2 cm monolayer MoS_2_ film grown by chemical vapor deposition (CVD). The photogating effect induced by the formation of trap states ensures a high photoresponsivity of 364 AW^−1^, which is considerably superior to traditional CMOS sensors (≈0.1 AW^−1^). Experimental results also show highly uniform photoelectric properties in this array. Finally, the concatenated image obtained by laser lighting stencil and photolithography mask demonstrates the promising potential of 2D MoS_2_ for future optoelectrical applications.

## 1. Introduction

Two-dimensional (2D) transition metal dichalcogenides (TMDs) have been developing rapidly and have received considerable research attention in the field of photodetection because of their superior electrical and optical properties [1,2,3,4]. Tremendous efforts have been dedicated to developing high-performance 2D TMDs-based photodetectors for potential applications in optical imaging, neural network vision sensor, and bioinspired in-sensor vision [5,6,7,8,9]. Molybdenum disulfide (MoS_2_), the most famous representative in the TMD family, has been extensively investigated for electronic and optoelectronic device applications owing to its unique properties, including the layer-dependent bandgap (1.8~1.2 eV from monolayer to bulk), relatively high electron mobility, and current on/off ratio (up to 10^9^) [10,11,12,13]. Therefore, MoS_2_ has been considered a promising channel material for low-power logic devices [14,15,16] and photodetectors in the visible range [17]. Most previously reported results were based on isolated MoS_2_ flakes obtained via widely-used top-down approaches such as mechanical exfoliation [18,19]. Such exfoliated single crystalline flakes provide good performance for a single fabricated device, but their micro-scale flake sizes and randomly distributed thicknesses also result in low yield and reproducibility, which hinder practical device applications [18,20].

In recent years, the large-scale fabrication of MoS_2_ devices has become mainstream since various wafer-scale bottom-up growth methods have been developed, such as CVD, atomic layer deposition (ALD), and metal-organic CVD (MOCVD). For example, Peng et al. presented CVD-grown MoS_2_ phototransistors with a high photoresponsivity of 6650 AW^−1^ and detectivity of 1.23 × 10^12^ [21]. Chu et al. produced a molybdenum-based phototransistor with an ultrasensitive detectivity of 9.8 × 10^16^ cm Hz^1/2^ W^−1^ [22]. Guo et al. reported the optoelectrical performances of stacked ML-MoS_2_ phototransistors [23]. In addition to the film fabrication, various device structures have been proposed to improve the performance of MoS_2_ photodetectors. Chen et al. presented a bilayer MoS_2_/graphene heterostructure array with the photoresponsivity of 32 mAW^−1^ [24]. Jeong et al. demonstrated a periodically arrayed nanopore structures for improving the efficiency of multilayered p-WSe_2_/n-MoS_2_ phototransistors with a photoresponsivity of 1.7 × 10^4^ AW^−1^ [25]. However, none of the above focused on the homogeneity of large-scale 2D TMD photodetector arrays, which is a key to realize a practical image sensor. Recently, several reported results tackled the fabrication of pixel array MoS_2_ image sensors. Park et al. reported a 4 × 4 multilayer MoS_2_ phototransistors array grown using a post-sulfurization process, which gives rise to a high uniformity but at the cost of a relatively low photoresponsivity of 3.7 AW^−1^ [26]. Hong et al. designed an 8 × 8 active pixel image sensor array based on a bilayer MoS_2_ film with a maximum photoresponsivity of 119.16 AW^−1^ [27]. In these works, how to maintain a uniform high performance while increasing the scale of the pixel array still remains an unsolved problem. In this study, we presented a 12 × 12 pixel array image sensor built on a 2 cm × 2 cm monolayer MoS_2_ film. Compared with previous work, all 144 individual pixels exhibit the desired optoelectrical properties (photoresponsivity of 364 AW^−1^, photo detectivity of 2.13 × 10^10^) with a high uniformity. Moreover, the MoS_2_ image sensor, placed under photolithography masks, was exposed to lights of visible-band wavelengths from a laser emitter. By illumination with a different wavelength and different stencils, three sets of photocurrent data were collected and converted to a visualization image, respectively. Thus, this work introduces a new platform for optoelectronic application of wafer-scale 2D-TMDs such as ultra-thin image sensors, transparent image sensors, artificial intelligence photo sensors, and selective light-detecting imagers.

## 2. Materials Synthesis and Characterizations

**Figure 1:**
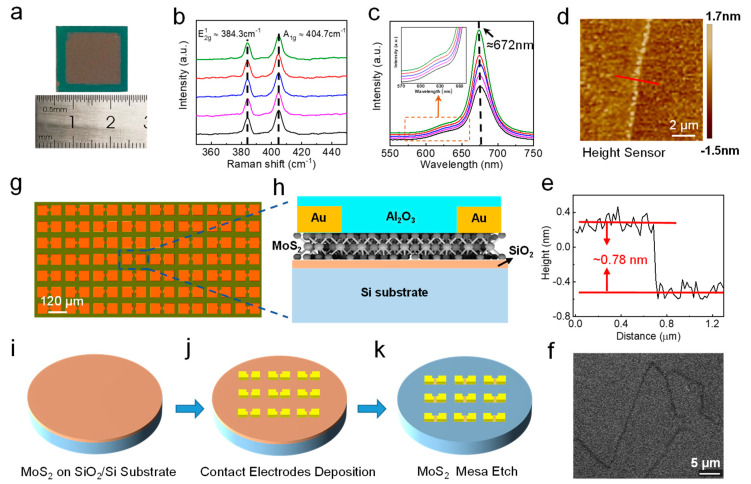
Spectroscopic analysis of the CVD-grown monolayer MoS_2_ film and process of the device structure. (**a**) Photograph of an as-fabricated centimeter scale MoS_2_ with FETs. (**b**) Raman spectra curves of five randomly selected points from the film, (**c**) PL spectra curves of five points with the same positions from the film, (**e**) thickness scan along the red line across the boundary of the film. (**d**) AFM image of the monolayer MoS_2_ film. (**f**) SHG mapping in an area of 30 μm × 30 μm of the film, scale bar: 5 μm. (**g**) Optical microscopy image of the as-fabricated 12 × 12 MoS_2_ phototransistors array. The scale bar is 120 μm. (**h**) 3D schematic image of a single MoS_2_-based phototransistor. (**i**–**k**) diagram for the fabrication process of MoS_2_ film to phototransistors devices employing back metal gates device structure.

Our pixel array image sensor was formed using a 2 cm × 2 cm CVD-grown monolayer MoS_2_ film, as shown in Figure 1a. This film was synthesized directly on a cleaned silicon oxide (SiO_2_) substrate without any transfer processes [28]. Compared with the transferring method, the CVD growing method does not use complex processes, implying a higher productivity and lower cost. More details on the film growth are provided in the Supplementary Materials. Prior to the device fabrication, multiple material characteristics, Raman spectra, photoluminescence (PL) spectra, atomic transmission microscope (AFM), and SHG were tested, and the corresponding results were given in Figure 1. In Figure 1b, the Raman spectra under the irradiation of a 514 nm laser were obtained from five different locations on the MoS_2_ film. The difference between two dominant peaks, i.e., the in-plane (*E*^1^_2g_) vibration mode at ~384.3 cm^−1^ and the out-of-plane (*A*_1g_) vibration mode at ~404.7 cm^−1^ was around 20 cm^−1^, which was consistent with previous work [18]. Moreover, the prominent consistency and nonexistence of splitting of all Raman spectra curves indicated the high uniformity of our CVD-grown MoS_2_ film. Figure 1c demonstrated the PL spectra from five suspended samples excited with a solid-state laser at a wavelength of 514 nm. A low laser power of 50 μW (on the sample) was used to avoid heating and PL saturation. The peak value at ≈1.84 eV in the PL spectra was a signal from the MoS_2_ with silicon substrate [28]. Furthermore, the near-identical peak positions of all curves validated the wafer-scale uniformity of the film as well. The height profile of MoS_2_ film was measured by AFM (Bruker Dimension Edge). The average height difference along the red line, as shown in Figure 1e, was around 0.78 nm, which corresponded exactly to the thickness of monolayer MoS_2_ film [11]. Since the information on domain size and boundaries was missing through AFM, a second harmonic generation (SHG) technique was applied to reveal more detailed morphology. As shown in Figure 1f, the domain size was about 10–20 µm in our monolayer, MoS_2_ and the grain boundaries could be clearly recognized from SHG images. Such capability of direct visualization of the grain information in MoS_2_ is attributed to the suppressed SHG signal at the grain boundaries. The difference in crystal orientations resulted in the destructive interference and annihilation of the nonlinear waves [29,30]. Above all, all the measurement results indicated the excellent uniformity of our CVD-grown film, which is crucial for further MoS_2_ phototransistor device fabrication.

The mature back-gate phototransistor structure was adopted here, and the detailed process is presented in Figure 1. The optical microscopic image of the pixel array photodetector with a large-area monolayer MoS_2_ film is demonstrated in Figure 1g. The device was composed of 12 × 12 MoS_2_ phototransistors with the same geometric size. A more detailed single device structure was illustrated in Figure 1h. Each phototransistor consists of a MoS_2_ channel with W/L of 30 μm/20 μm and Au electrodes for source and drain contacts. Figure 1i–k displays a schematic fabrication flow of our MoS_2_ pixelarray image sensor. As described before, the monolayer MoS_2_ film was grown on a SiO_2_/Si substrate, Au (35 nm), as the source/drain (S/D) electrodes were deposited using an electron-beam evaporator and photolithography via a lift-off technique, and then the channel isolation was realized by the CF_4_ reactive ion etching using a photoresist mask, and a MoS_2_ channel was etched to the designated dimensions. Finally, Aluminum oxide (40 nm) was deposited as the protective insulator via ALD. The details of the device fabrication are presented in the Supplementary Information.

Compared with the top-gate phototransistor structure, the back gate structured phototransistor can absorb light more efficiently. Moreover, MoS_2_ is directly grown on a SiO_2_/Si substrate without additional processing (e.g., transferring MoS_2_ onto a glass or flexible substrate [31]), which also contributes to a higher uniformity and lower fabrication cost.

## 3. Results and Discussion

**Figure 2:**
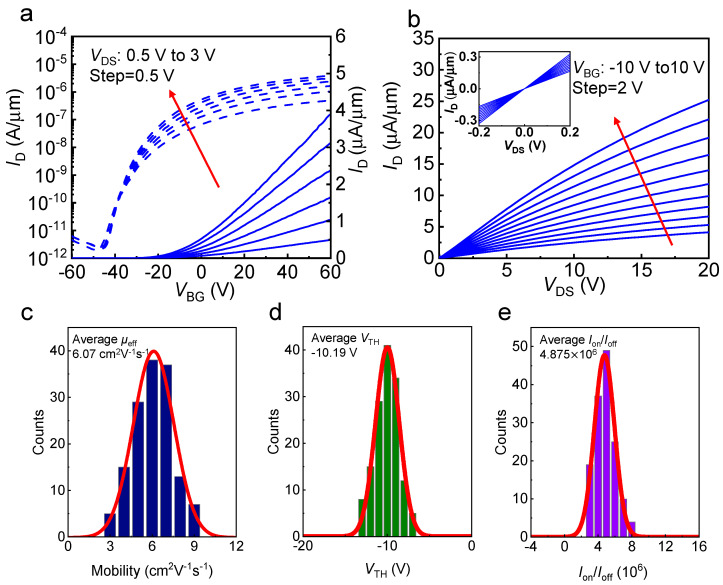
Electrical characteristics and statistical analysis of MoS_2_ phototransistors. (**a**) *I*_D_-*V*_BG_ curves of a typical MoS_2_ phototransistor at *V*_DS_ from 0.5 to 3 V with the step of 0.5 V. (**b**) *I*_D_-*V*_DS_ curves of MoS_2_ at *V*_BG_ from −10 to 10 V with the step of 2 V. Inset: *I*_D_-*V*_DS_ curve acquired at a small range of *V*_DS_. The Histograms of (**c**) field-effect mobility (average *μ*_eff_ = 6.07 cm^2^ V^−1^ s^−1^), (**d**) threshold voltage (average *V*_TH_ = −10.19 V), and (**e**) *I*_on_/*I*_off_ current ratio (average *I*_on_/*I*_off_ = 4.875 × 10^6^) with *V*_DS_ = 1 V of all 12 × 12 MoS_2_ phototransistor pixels.

Then, we tested the electrical properties of as-fabricated MoS_2_ phototransistors. The typical transfer characteristics (*I*_D_-*V*_BG_) of a single MoS_2_ phototransistor was plotted in Figure 2a. The current on/off ratio (*I*_on_/*I*_off_) of ≈10^6^ and the threshold voltage (*V*_TH_) of −9.2 V at a drain voltage (*V*_DS_) of 1 V indicated the strong gate modulation of the designed MoS_2_ device. Figure 2b displayed the output characteristics of a typical MoS_2_ phototransistor in the pixel array image sensor. The drain current (*I*_D_) was saturated at a high drain bias (>20 V) because of pinch-off at the drain region. Due to a satisfied contact formation between the monolayer MoS_2_ film and the S/D electrodes (Au), the output characteristics, *I*_D_-*V*_DS_, in the insets of Figure 2b exhibited a linear behavior of *I*_D_ at a low drain bias [32]. Field-effect carrier mobility *μ*_eff_ could also be extracted from the linear region of the transfer curve using the following equation $\mu_{\mathrm{eff}}=g_{m}\frac{L_{c}}{W_{c}C_{\mathrm{ox}}V_{\mathrm{DS}}}$, where *g*_m_ is the transconductance, *L*_c_ and *W*_c_ are the length and width of the channel, respectively, *C*_ox_ is the capacitance of the gate insulator, and *V*_DS_ is the drain voltage [33].

To further demonstrate the uniformity of our image sensor in terms of electrical properties, histogram and corresponding fitted Gaussian distribution were also calculated for mobility (*μ*_eff_), threshold voltage (*V*_TH_), and on/off current ratio (*I*_on_/*I*_off_) measured from all 12 × 12 phototransistors, as summarized in Figure 2c–e, respectively. The corresponding fitted Gaussian curves (the red solid lines) were also plotted for each parameter. All the phototransistors exhibited a satisfactory electrical performance with the following average values: a *μ*_eff_ of 6.07 cm^2^ V^−1^ s^−1^, a *V*_TH_ of −10.2 V, and an *I*_on_/*I*_off_ of 4.87 × 10^6^. According the equation $STD\left(D\right)=\sqrt{\frac{\sum_{i=1}^{144}{\left(D_{i}-\bar{D}\right)}^{2}}{144}}/\bar{D}$, where *D* could be replaced by *μ*_eff_, *V*_TH_ and *I*_on_/*I*_off_. The standard deviations (*STD*) of carrier mobility *μ*_eff_ is 23%. The *STD* of threshold voltage *V*_TH_ and current ratio *I*_on_/*I*_off_ are 2.8% and 25.2%, respectively. Compared with previous works [26,27], our image sensor exhibited better electrical performance and much smaller pixel-level variation with a much greater number of phototransistors.

**Figure 3:**
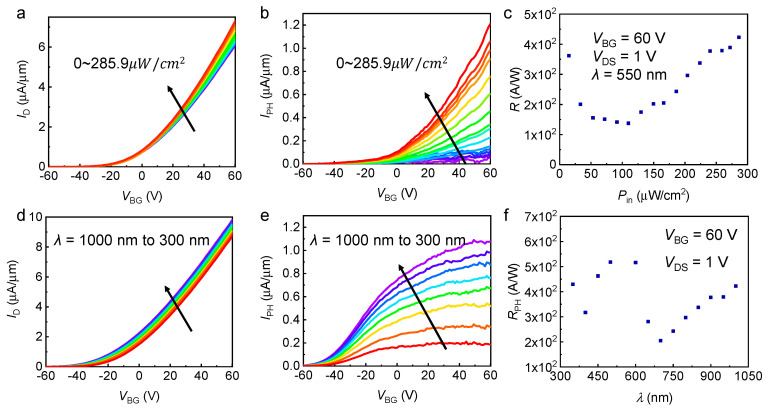
Photo-responsive characteristics of a monolayer MoS_2_ phototransistor in the image sensor array. (**a**) Transfer curves of *I*_D_ for dark and illumination conditions and (**b**) Photocurrent as the function of *V*_BG_ when *V*_DS_ = 1 V with varying lighting intensity, ranging from 14.7 to 285.9 μW/cm^2^ with an average step of 17.87 μW/cm^2^. (**c**) Responsivity as a function of incident power when *V*_BG_ = 60 V and *V*_DS_ = 1 V. (**d**) Transfer curves for illumination conditions with varying light wavelengths. (**e**) Photocurrent as a function of *V*_BG_ when *V*_DS_ = 1 V with different wavelengths, ranging from 1000 nm to 300 nm with a step of −100 nm. (**f**) Responsivity as a function of the light wavelength.

In addition to the electrical characteristics, the key opto-electric characteristics of a single MoS_2_ phototransistor were measured in Figure 3. Figure 3a exhibited the photoinduced transfer characteristics *I*_D_-*V*_BG_ for a typical MoS_2_ photodetector 550 nm illumination wavelength at given incident power densities (*P*_in_) ranging from 14.7 to 285.9 μW/cm^2^. The photocurrent curve of Figure 3b was further extracted from Figure 3a by the following equation: *I*_PH_ = *I*_illumination_ − *I*_dark_. The photocurrent of the monolayer MoS_2_ phototransistor gradually increased with increasing *P*_in_ according to the photocurrent value under light with different power densities. Moreover, the photoinduced transfer characteristics and photocurrent curve under light with changeable excitation wavelength are presented in Figure 3d,e. Monolayer MoS_2_ is more sensitive to visible wavelength band (420–680 nm) compared with near-infrared band (700–1200 nm). Figure 3c,f displayed the curve of the calculated photoresponsivity *R*_PH_ as a function of incident power density and excitation wavelength, which were important figures of merit for phototransistors. The *R*_PH_ was extracted from the transfer characteristics in Figure 3b,e using the equation of *R*_PH_ = *I*_PH_/*P*_in_ (unit: AW^−1^), where *I*_PH_ and *P*_in_ are the photocurrent and incident power density, respectively. The main mechanism for the high *R*_PH_ of the monolayer MoS_2_ phototransistor is the photogating (PG) effect by the formation of trap states near the valance band due to the structural imperfection and defects of MoS_2_ [34,35,36,37].

**Figure 4:**
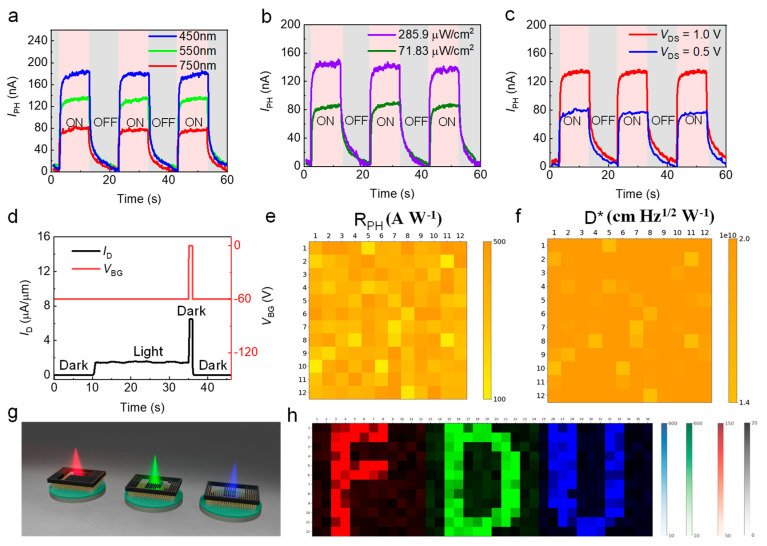
(**a**) Time-trace of the photodetector under illumination with the same intensity (*P*_in_ = 200 μW/cm^2^) and *V*_DS_ = 1 V but at different wavelengths (*λ* = 450, 550 and 750 nm). (**b**) Same measure for different light intensities (*P*_in_ = 285.9 μW/cm^2^ and 71.83 μW/cm^2^) with the wavelength of 550 nm and *V*_DS_ = 1 V. (**c**) Same measurement for different *V*_DS_ values (0.5 and 1.0 V) under the illumination of 285.9 μW/cm^2^ with 550 nm wavelength. (**d**) Photo-switching characteristics with gate pulse. (**e**,**f**) Photo-responsivity and detectivity mapping of 144 MoS_2_ phototransistors under the illumination of 285.9 μW/cm^2^ with 550 nm wavelength. (**g**) Measurement concept using the light stencil projection for image detection of the image sensor array. The 12 × 12 monolayer MoS_2_ image sensor array is placed behind character masks and measured photoelectricity under RGB light illumination (wavelength *λ* = 750, 550, and 450 nm). (**h**) Horizontal concatenated normalized image with a resolution of 36 × 12 pixels.

Finally, Figure 4 presents the photo-response speed and an overall uniformity of the 12 × 12 MoS_2_ array. Figure 4a showed its switching behaviors under pulsed RGB light illumination. As it can be noticed, the photoresponsivity under 450 nm laser was much larger but with a slower response than that of the 750 nm laser. The rise time and decay time increased as light wavelength decreases due to a more excited number of photogenerated charge carriers [36,38]. Figure 4b showed a light intensity dependence, in which the *I*_PH_ and response time are all positively correlated to incident power, which is also attributed to the photogating effect [17]. Figure 4c presents the photo-switching behavior of *I*_PH_ under different *V*_DS_ values. The photoresponsivity increases at the expense of a longer response time. The switching characteristics under different back-gate voltages (*V*_BG_) are displayed in Figure S1. When *V*_BG_ was over-high (>10 V) or over-low (<−10 V), the recovery time would be longer for photo-excited carriers to detrap from the subgap state [27], whereas the gate pulse method by changing back-gate voltage *V*_BG_ would improve greatly the response speed of our image sensor array. As shown in Figure 4d, when the illumination switched from light to dark, the *V*_BG_ was injected simultaneously with a short-time (≈1 s) pulse voltage from 8 V to 15 V to suppress carriers as soon as possible. As a result, the falling time decreased by 90% compared with Figure 4a–c. A more detailed gate pulse effect was displayed in Figure S2 and Table S1. A short duration gate voltage pulse could reduce the decay time significantly due to the detrapping of the trapped holes in subgap states, which enables the high-speed operation of the MoS_2_ image sensor [27,39].

To demonstrate the overall uniformity of our image sensor, a statistical distribution of the photoelectrical properties, i.e., responsivity and detectivity of 12 × 12 MoS_2_ phototransistors was confirmed in the mapping images that presented the current level of each phototransistor under illumination with *P*_in_ of 285.9 μW/cm^2^ (Figure 4e,f), respectively, where all currents were measured at a *V*_DS_ of 1 V. The detectivity (D^*^) is obtained by the equation of $D^{*}=\sqrt{\frac{A}{2qI_{D}}}R_{\mathrm{PH}}$, where *A* is the channel area, *q* is unit electric charge, *I*_D_ is dark current, and *R*_PH_ is photoresponsivity. The average photoresponsivity was 364.00 AW^−1^ with a standard deviation of 99 AW^−1^. The detectivity was 2.16 × 10^10^ cm Hz^1/2^ W^−1^ with a standard deviation of 3.23 × 10^9^ cm Hz^1/2^ W^−1^. The photocurrent maps under illumination with different wavelengths (red: 750 nm, green: 550 nm, blue: 450 nm) are demonstrated in Figure S3, which also proves the uniformity of our device. 

Moreover, three sets of masks (each was 12 × 12 pixels) with different characters (“F”, “D” and “U”) were prepared and patterned using a laser cutting system, as shown in Figure 4g, to evaluate the image-sensing characteristics of the monolayer MoS_2_ image sensor array. These character masks were sequentially placed on the image sensor array during light projection (red: 750 nm, green: 550 nm, blue: 450 nm). The 2D photocurrent data, collected under light stencil, was firstly normalized from float type to then concatenated horizontally to form the final 36 × 12 image (total pixel: 576). Due to the uniform photo-related properties of all 12 × 12 phototransistors, the photosensitivity mapping result could display three characters clearly. Table 1 compared the fundamental properties with former works.

## 4. Conclusions

In this paper, a 12 × 12 phototransistor pixel array image sensor based on a wafer-scale monolayer MoS_2_ film was fabricated to present the potential of the next generation photodetector. The integrated pixel number is significantly increased without sacrificing photodetector performance. The fabricated MoS_2_ devices showed high uniformity in electrical properties, including carrier mobility (≈6.07 cm^2^ V^−1^ s^−1^), *I*_on_/*I*_off_ (≈4.875 × 10^6^), and threshold voltage *V*_TH_ (≈−10.19 V). The measured photoresponsivity *R*_PH_ (≈364 AW^−1^) and detectivity (≈2.13 × 10^10^) were superior to traditional CMOS image sensors [40,41], which were attributed to the predominant photogeneration mechanism of the PG effect induced by the formation of trap states near the valance band due to the structural imperfection and defects of MoS_2_. These results provide a blueprint for the future development of wafer-scale 2D TMD optoelectrical application and suggest further application scenarios requiring a high dynamic range, such as with artificial retinas.

## Figures and Tables

**Table 1:** Comparison of photo-related properties of fabricated pixel array image sensors.

Indicator	Park et al. [26]	Hong et al. [27]	Ours
Pixel size (width × height)	4 × 4	8 × 8	12 × 12
Layer of MoS_2_ film	2 L	Multilayer	1 L
Average responsivity (Unit: A W^−1^)	0.503	119.16	364.00
Std responsivity (Unit: percentage %)	15	--	27.2
Average detectivity (Unit: cm Hz^1/2^ W^−1^)	1.4 × 10^4^	4.66 × 10^6^	2.13 × 10^10^
Std detectivity (Unit: percentage %)	12	--	15

## Data Availability

The data presented in this study are available on request from the corresponding author.

## Supplementary Materials

The following supporting information can be downloaded at: https://www.mdpi.com/article/10.3390/nano12234118/s1, Figure S1: Photoswitching characteristics of the MoS_2_ phototransistor under temporal light illumination with varying back gate voltage *V*_BG_ from −30 V to 30 V with a step of 10 V. All switching curves were measured at *V*_DS_ = 1 V with illumination frequency of 0.05 Hz; Figure S2: A Time resolved photoresponsive characteristics of the monolayer MoS_2_ phototransistor under temporal light illumination with λ = 550 nm without and with gate voltage pulse. The fall time is improved from 7.59 s to 6.24 s; Figure S3: Photocurrent mapping of 12 × 12 MoS_2_ phototransistors at *V*_DS_ = 1 V, *V*_BG_ = −10 V under RGB light illumination (λ = 750, 550, and 450 nm), indicating uniform photocurrent photoresponses; Table S1: The rise time and fall time with different case. Reference [42] was cited in supplementary materials.
