# Supplementary material for: Large-Scale MoS2 Pixel Array for Imaging Sensor
Source: Nanomaterials (Basel). 2022 Nov 22;12(23):4118. doi: 10.3390/nano12234118 (PMC9739261; doi:10.3390/nano12234118)
Supplement: Supplementary file 1 [file nanomaterials-12-04118-s001.zip › nanomaterials-2028436-supplementary.pdf]

nanomaterials

Supplementary Materials

Large-Scale MoS_2 Pixel Array for Imaging Sensor

Kang Liu [1,†], Xinyu Wang [1,†], Hesheng Su [1], Xinyu Chen [1], Die Wang [1], Jing Guo [1], Lei Shao [2,*], Wenzhong Bao [1,*] and Honglei Chen [3,*]

[1] State Key Laboratory of ASIC and System, School of Microelectronics, Zhangjiang Fudan International Innovation Center, Fudan University, Shanghai 200433, China
[2] School of Electronic Information, Soochow University, Suzhou 215006, China
[3] Shanghai Institute of Technical Physics, Chinese Academy of Sciences, Shanghai 200083, China
* Correspondence: shaolei@suda.edu.cn (L.S.); baowz@fudan.edu.cn (W.B.); chenhl@mail.sitp.ac.cn (H.C.)
† These authors contributed to this work equally.

Materials Preparation

The monolayer MoS_2 film synthesized on a silicon substrate was growth in a two-temperature-zone CVD chamber. The adequate sulfur powder (Alfa Aesar, 99.999%) and MoO_3 powder (Alfa Aesar 99.95%), used as material sources for film fabrication, were loaded in two separate inner tubes and placed at different zones by a distance of 30 cm [1]. During the growth, the two inner tubes were flowed with Ar as carrying gases, and the growth temperature for the sulfur and MoO_3 precursors was controlled at 180 and 650 °C, respectively [2]. The continuous monolayer MoS_2 film was synthesized at room pressure with 15 min sulfuration time.

Device Fabrication

A conventional photolithography laser direct writing (MicroWriter ML③3) is performed to form the source and drain electrodes pattern and then 35 nm Au was deposited by E-beam evaporation, next lift-off the excess metal, followed by second lithography and CF_4 reactive ion etching to define the channel region. Finally, Aluminum oxide (40 nm) was deposited as the protective insulator via ALD.

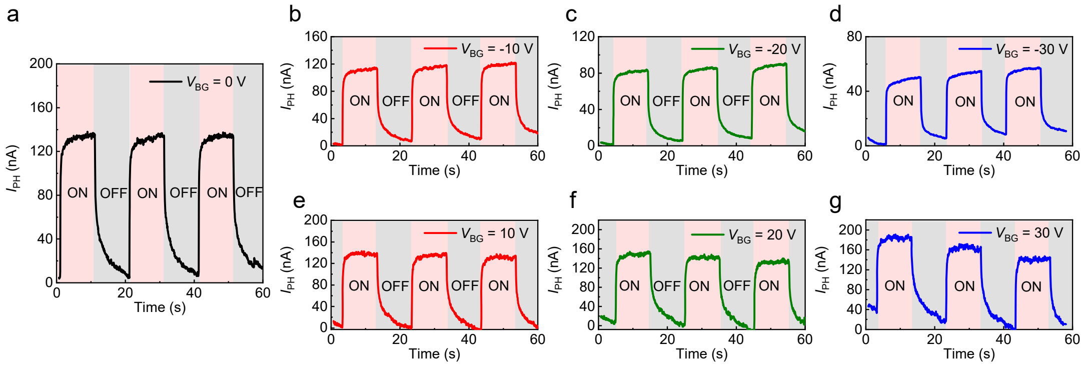

Figure S1. Photoswitching characteristics of the MoS_2 phototransistor under temporal light illumination with varying back gate voltage V_{BG} from -30 V to 30 V with a step of 10 V. All switching curves were measured at V_{DS} = 1 V with illumination frequency of 0.05 Hz.

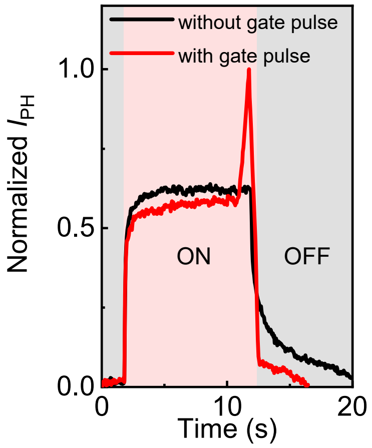

Figure S2. A Time resolved photoresponsive characteristics of the monolayer MoS_2 phototransistor under temporal light illumination with λ = 550 nm without and with gate voltage pulse. The fall time is improved from 7.59 s to 6.24 s.

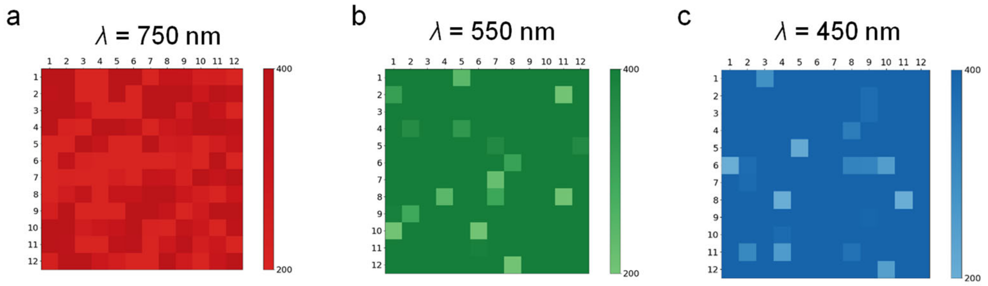

Figure S3. Photocurrent mapping of 12 × 12 MoS_2 phototransistors at V_{DS} = 1 V, V_{BG} = −10 V under RGB light illumination (λ = 750, 550, and 450 nm), indicating uniform photocurrent photoresponses.

Table S1. The rise time and fall time with different case.

		**Rise time (s)**	**fall time (s)**
Wavelength	Red (750nm)	2.8	2.5
	Green (550nm)	1.8	1.6
	Blue (450nm)	2.4	2.2
Illuminance	Strong (285 μW/cm^2)	2.1	1.7
	Weak (71μW/cm^2)	2.4	2.1
V_{DS}	1.0V	1.9	1.7
	0.5V	2.2	2.0

References

1. Xu, H.; Zhang, H.; Guo, Z.; Shan, Y.; Wu, S., Wang, J. High-performance wafer-scale MoS_2 transistors toward practical application. *Small* **2018**, *14*, 1803465.
2. Yu, H.; Liao, M.; Zhao, W.; Liu, G.; Zhou, X.J.; Wei, Z.; Xu, X.; Liu, K.; Hu, Z.; Deng, K.; et al. Wafer-Scale Growth and Transfer of Highly-Oriented Monolayer MoS_2 Continuous Films. *ACS Nano* **2017**, *11*, 12001–12007, https://doi.org/10.1021/acsnano.7b03819.
